# Impact of Delayed Recovery of Independent Ambulation and Sarcopenia Progression on Long‐Term Outcomes Following Endovascular Aortic Aneurysm Repair

**DOI:** 10.1111/ggi.70355

**Published:** 2026-01-21

**Authors:** Hirokazu Sugiura, Tsuyoshi Shibata, Yutaka Iba, Shingo Tsushima, Kenta Yoshikawa, Shun Hayasaka, Tomohiro Nakajima, Junji Nakazawa, Ayaka Arihara, Kenichi Kato, Shigeki Komatsu, Masato Yonemori, Hajime Maeda, Masanori Nakamura, Yuki Sugawara, Nobuyoshi Kawaharada

**Affiliations:** ^1^ Department of Rehabilitation Sapporo City General Hospital Sapporo Japan; ^2^ Department of Surgery, Division of Cardiovascular Surgery Sapporo Medical University Sapporo Japan; ^3^ Department of Cardiovascular Surgery Sapporo Teishinkai Hospital Sapporo Japan; ^4^ Division of Radiology and Nuclear Medicine Sapporo Medical University Sapporo Japan; ^5^ Department of Cardiovascular Surgery Sapporo City General Hospital Sapporo Japan; ^6^ Department of Radiology Sapporo City General Hospital Sapporo Japan

**Keywords:** ambulation, aortic aneurysm, prognosis, psoas muscles, sarcopenia

## Abstract

**Aim:**

To evaluate the long‐term prognostic impact of delayed recovery of independent ambulation and post‐operative sarcopenia progression in patients undergoing endovascular aortic aneurysm repair (EVAR).

**Methods:**

In this multicenter retrospective cohort study, 228 patients (mean age 78.1 ± 6.5 years; 82.5% male) who underwent EVAR for abdominal aortic aneurysm between January 2015 and December 2020 were included. Independent ambulation was defined as walking ≥ 15 m. Sarcopenia was assessed using the psoas muscle index (PMI) at L3 on CT, normalized by height squared. Baseline PMI was measured within 3 months preoperatively; post‐operative sarcopenia progression was calculated as ΔPMI/baseline (% change from baseline to 6 months). The primary outcome was all‐cause mortality, analyzed using multivariate Cox proportional hazards models.

**Results:**

Over a mean follow‐up of 4.6 ± 2.2 years, 52 patients (22.8%) died. Mean time to independent ambulation was 1.4 ± 1.2 days, and mean ΔPMI/baseline decreased by 4.5% ± 8.9%. After adjusting for age, sex, nutritional status, and pre‐operative sarcopenia, time to independent ambulation (HR 1.25; 95% CI 1.07–1.46; *p* = 0.004) and ΔPMI/baseline (HR 1.13; 95% CI 1.09–1.17; *p* < 0.001) were independent predictors of mortality. ROC analysis identified cut‐offs of ≥ 2 days for ambulation and a decrease of ≥ 6.09% in ΔPMI/baseline. Patients meeting both criteria exhibited the poorest survival, representing delayed ambulation and marked sarcopenia progression.

**Conclusions:**

Delayed recovery of independent ambulation and post‐operative sarcopenia progression independently predict all‐cause mortality after EVAR and may serve as clinically useful indicators for risk stratification and targeted rehabilitation.

## 1 Introduction

Since its initial clinical implementation in 1991, endovascular aortic aneurysm repair (EVAR) has been broadly adopted globally as a main intervention for abdominal aortic aneurysms (AAA). Currently, EVAR constitutes approximately 80% of AAA treatments and has become the standard therapeutic strategy [1]. Compared with open surgery, EVAR provides superior short‐term prognosis, particularly in terms of 30‐day mortality and perioperative complications. However, its higher long‐term mortality rate remains a major concern [2, 3]. As EVAR is a minimally invasive procedure associated with significantly less intraoperative stress response, blood loss and post‐operative pain as well as a shorter hospital stay than open surgery, the significance of evaluating the changes in muscle status and impact on activities of daily living following surgery is frequently underestimated and overlooked [4].

Conversely, patients eligible for EVAR are typically frail older adult patients or those with compromised general health, including those with multiple comorbidities [5]. Therefore, delayed recovery of independent ambulation is anticipated in a larger proportion of patients following EVAR. Previous studies in patients undergoing cardiac surgery have identified delayed recovery of independent ambulation as an independent predictor of poor prognosis [6]. However, the association between delayed recovery of independent ambulation and clinical outcomes in patients undergoing EVAR remains uninvestigated.

Recent studies have noted a significant post‐operative reduction in psoas muscle area (PMA) on computed tomography (CT) following EVAR compared with thoracic endovascular aortic repair, another minimally invasive procedure. Moreover, EVAR has been identified as an independent risk factor for psoas muscle atrophy. Ischaemic injury to the psoas muscle during EVAR has been suggested as a possible mechanism underlying this decreased muscle volume [4]. Several studies have identified post‐operative sarcopenia progression following major abdominal and thoracic surgeries as a prognostic factor associated with poor outcomes [7, 8]. Although pre‐operative sarcopenia has been recognized as a negative prognostic indicator in patients undergoing EVAR [9], studies investigating the association between post‐operative sarcopenia progression and patient prognosis are lacking.

We hypothesized that delayed recovery of independent ambulation and sarcopenia progression following EVAR can adversely influence long‐term prognosis. To the best of our knowledge, this is the first study to evaluate the combined impact of two previously underexplored factors, including delayed recovery of independent ambulation and post‐operative sarcopenia progression, on long‐term outcomes following EVAR. Therefore, this study aimed to evaluate the impact of their prognostic significance in this patient population.

## 2 Methods

### 2.1 Study Design and Patient Selection

This retrospective observational multicenter study was conducted at two centers in Japan. Patients who underwent EVAR at Sapporo City General Hospital and Sapporo Medical University from January 2015 to December 2020 were included (*n* = 292). The following were the exclusion criteria: aged < 65 years, emergency surgery, impaired independent ambulation before surgery, without follow‐up CT at 6 months, re‐treatment during the follow‐up period and transferred to another medical department. The decision to perform EVAR rather than open surgery was made at the discretion of a vascular surgeon‐led multidisciplinary team. For elective cases, EVAR was generally preferred for older patients with favorable anatomical characteristics. Anatomic feasibility was assessed on the basis of the length and morphology of the aneurysm neck, as well as the accessibility of the iliac arteries.

### 2.2 End Points

All‐cause mortality over the follow‐up period was the primary end point, and the incidence of major adverse cardiovascular events (MACEs) during the follow‐up period was the secondary end point. MACEs were defined as stroke, heart failure, myocardial infarction, aortic aneurysm‐related events (e.g., aortic dissection or aortic rupture), and cardiovascular mortality. Clinical events were ascertained from medical records. Patient follow‐up was conducted via telephone interview in cases where medical records were unavailable. Complete follow‐up data following EVAR were collected whenever possible.

### 2.3 Definitions

Post‐operative rehabilitation was provided to all patients by physical therapists or specialized nursing staff, following protocols and progression criteria based on the JCS/JACR 2021 guidelines for cardiovascular rehabilitation [10]. All patients received rehabilitation under these standardized protocols, ensuring uniform assessment of functional recovery. In this study, independent ambulation was defined as the ability to walk at least 15 m unaided, in accordance with the Functional Independence Measure criteria for independent indoor mobility [11]. Sarcopenia was assessed using the psoas muscle index (PMI), calculated as the cross‐sectional area of the PMA at the third lumbar vertebra level on pre‐operative CT images divided by height squared [12]. PMI was selected because the PMA can be measured easily without the need for specialized image analysis software or automated algorithms, and its measurement is highly reproducible. Pre‐operative CT images were obtained within 3 months pre‐operatively. The slice was selected at the level of the third lumbar vertebra, where the lateral tips of both transverse processes were visible (Figure S1) [13, 14]. The slice thickness was 3–5 mm. The left and right PMAs were manually delineated and quantified using SYNAPSE (Fujifilm Medical Corporation, Tokyo, Japan) and the total area was subsequently calculated on the basis of muscle contours visualized in cross‐sectional images. A single investigator who was blinded to patient outcomes performed all measurements. According to the diagnostic criteria in Asian adults, pre‐operative sarcopenia was defined as a PMI of < 6.36 cm^2^/m^2^ in males and 3.92 cm^2^/m^2^ in females [12]. This cut‐off has also been applied in other Asian cohorts and has been shown to be associated with adverse post‐operative outcomes [15, 16]. PMI change (ΔPMI) was defined as the difference between the pre‐operative PMI (baseline) and the PMI at 6 months post‐operatively. Post‐operative CT images were obtained as part of routine follow‐up. The degree of post‐operative sarcopenia progression was expressed as ΔPMI/baseline.

### 2.4 Data Collection

Baseline characteristics included age, sex, body mass index (BMI), American Society of Anaesthesiologists (ASA) classification, AAA diameter, comorbidities, and the Geriatric Nutritional Risk Index (GNRI), which was calculated using the formula GNRI = (14.89 × serum albumin [g/dL]) + (41.7 × BMI/22), with lower values indicating higher nutritional risk [17]. Perioperative parameters comprised type of stent grafts, operative time, anesthesia time, intraoperative blood loss, need for transfusion, internal iliac artery embolization, inferior mesenteric artery embolization, post‐operative delirium incidence, time to independent ambulation, length of hospital stay, and rate of discharge to home.

### 2.5 Statistical Analysis

Continuous variables were presented as means ± standard deviations, whereas categorical variables were expressed as frequencies and percentages. Within‐group comparisons were performed using paired Student's *t*‐test. Between‐group comparisons were conducted using one‐way analysis of variance (ANOVA) for continuous variables and the chi‐square test for categorical variables. In addition, logistic regression analysis was performed for binary outcomes. The Kaplan–Meier method, log rank test, and Cox proportional hazards regression analysis were employed for assessing the primary and secondary end points. In the multivariate Cox proportional hazards regression analysis, variables that were significant in the univariate analysis, along with age and sex, were included as covariates. Optimal cut‐off values for significant variables associated with the primary end point in the Cox proportional hazards regression analysis were determined using receiver operating characteristic (ROC) curve analysis, with the Youden index used for identifying the point maximizing sensitivity and specificity. All statistical analyses were performed using EZR (Saitama Medical Centre, Saitama, Japan) [18], and *p* < 0.05 was considered statistically significant.

## 3 Results

### 3.1 Baseline Characteristics and Follow‐Up Results

**FIGURE 1:**
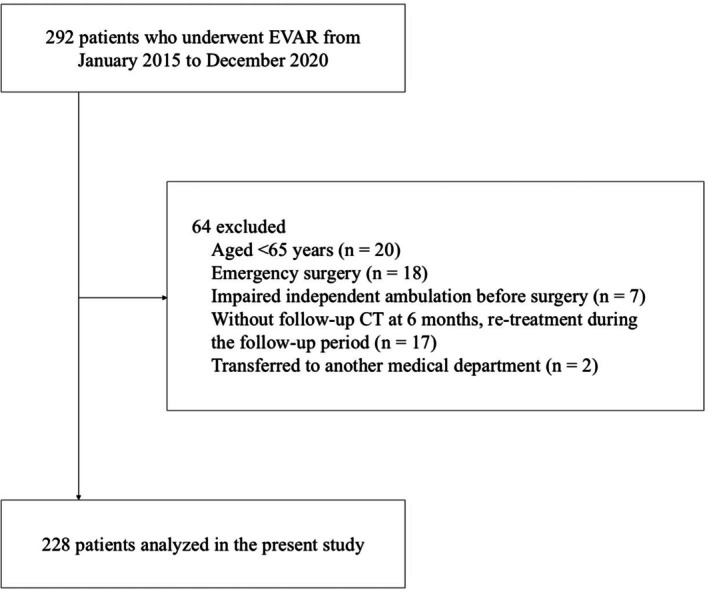
Flowchart of patient selection.

Following the exclusion of ineligible patients, 228 patients were included in the final analysis (Figure 1). The baseline characteristics and follow‐up results of the study population are summarized in Table 1. The mean age of the patients was 78.1 ± 6.5 years, 82.5% were male and 52.6% had an ASA physical status classification of ≥ 3. The mean AAA diameter was 49.8 ± 11.1 mm. During intervention, hypertension (68.0%) and smoking history (71.1%) were the most common comorbidities. The mean GNRI score was 102.8 ± 9.5. Post‐operative delirium occurred in 9.2% of the patients. The mean time to independent ambulation following surgery was 1.4 ± 1.2 days. Furthermore, 98.2% of the patients were discharged home and the mean post‐operative length of stay was 9.4 ± 5.4 days. Figure S2 presents a histogram illustrating the distribution of time to independent ambulation.

**TABLE 1:** Baseline characteristics and follow‐up results of the study population.

Variable	Patients (*n* = 228)
Demographics	
Age, years	78.1 ± 6.5
Male	188 (82.5)
Body mass index, kg/m^2^	23.6 ± 3.5
ASA score ≥ 3	120 (52.6)
AAA diameter, mm	49.8 ± 11.1
Comorbidities	
Hypertension	155 (68.0)
Dyslipidemia	83 (36.4)
Diabetes mellitus	39 (17.1)
Coronary artery disease	72 (31.6)
Peripheral arterial disease	15 (6.6)
Chronic obstructive pulmonary disease	34 (14.9)
Stroke or transient ischemic attack	44 (19.3)
Chronic kidney disease (eGFR < 60 mL/min/1.73m^2^)	50 (21.3)
Ever smoker	162 (71.1)
GNRI	102.8 ± 9.5
Perioperative parameters	
Type of stent grafts	
Excluder	165 (72.4)
Endurant	28 (12.2)
AFX	22 (9.6)
Other	13 (5.7)
Operation time, min	139.0 ± 54.6
Anesthesia time, min	204.8 ± 58.6
Intraoperative blood loss, mL	68.6 ± 119.9
Need for transfusion	7 (3.1)
IIA embolization	78 (34.2)
IMA embolization	26 (11.4)
Post‐operative delirium	21 (9.2)
Time to independent ambulation, days	1.4 ± 1.2
Length of hospital stay, days	9.4 ± 5.4
Rate of discharge to home	224 (98.2)
Follow‐up results	
Mean follow‐up period, years	4.6 ± 2.2
All‐cause mortality	52 (22.8)
3‐year survival rate, %	87.6 ± 2.3
5‐year survival rate, %	76.4 ± 3.2
MACEs	34 (14.9)

*Note:* Data are presented as *n* (%), mean ± standard deviation, or mean ± standard error for survival rates.

Abbreviations: AAA, abdominal aortic aneurysms; ASA, american society of anesthesiologists; eGFR, estimated glomerular filtration rate; GNRI, the geriatric nutritional risk index; IIA, internal iliac artery; IMA, inferior mesenteric artery; MACEs, major adverse cardiovascular events.

The mean follow‐up duration was 4.6 ± 2.2 years and the 3‐ and 5‐year survival rates were 87.6% ± 2.3% and 76.4% ± 3.2%, respectively. Of the 52 recorded mortalities, 12 (23.1%), 11 (21.2%), 10 (19.2%), 7 (13.4%), and 12 (23.1%) were due to cancer, cardiovascular causes, respiratory causes, cerebrovascular causes, and other causes, respectively. The incidence of MACEs during the follow‐up period was 34 patients (14.9%).

### 3.2 Psoas Muscle Index at Baseline and 6 Months Post‐Operatively

Changes in PMI between the baseline and 6‐month post‐operative CT are presented in Table 2. The mean baseline PMI was 4.86 ± 1.36 cm^2^/m^2^. Based on the sarcopenia criteria for Asian adults, 176 patients (76.9%) had pre‐operative sarcopenia, defined as a PMI < 6.36 cm^2^/m^2^ in males and < 3.92 cm^2^/m^2^ in females [12]. The PMI significantly decreased from baseline to 6 months post‐operatively (*p* < 0.001), with a mean ΔPMI/baseline decrease of 4.51% ± 8.94%. Figure S3 shows histograms of the distributions of PMI at baseline and ΔPMI/baseline.

**TABLE 2:** Psoas muscle index at baseline and 6 months post‐operatively.

Variable	Patients (*n* = 228)
PMI at baseline, cm^2^/m^2^	
Mean	4.86
SD	1.36
Minimum	1.63
Maximum	10.02
Pre‐operative sarcopenia^a^, *n* (%)	176 (76.9)
PMI at 6 months post‐operatively, cm^2^/m^2^	
Mean	4.64
SD	1.37
Minimum	1.63
Maximum	9.32
Difference (6 months post‐operatively minus baseline), cm^2^/m^2^	
Mean	−0.22
SD	0.41
*p*	< 0.001^b^
ΔPMI/baseline, %	
Mean	−4.51
SD	8.94

Abbreviations: BL, baseline; PMI, psoas muscle index; SD, standard deviation.

^a^ Pre‐operative sarcopenia was defined as a PMI below 6.36 cm^2^/m^2^ in men and 3.92 cm^2^/m^2^ in women.

^b^ Paired *t*‐test for identifying significant differences of mean values between baseline and 6 months after surgery CT images.

### 3.3 Univariate and Multivariate Cox Proportional Hazards Regression Analyses for All‐Cause Mortality and MACEs

The results of univariate and multivariate Cox proportional hazards regression analyses for all‐cause mortality and MACEs are summarized in Table 3. For all‐cause mortality, univariate analysis identified age, sex, BMI, AAA diameter, GNRI, time to independent ambulation, length of hospital stay, pre‐operative sarcopenia, and ΔPMI/baseline as significant factors. In multivariate analysis, time to independent ambulation (hazard ratio [HR], 1.25; 95% confidence interval [CI], 1.07–1.46; *p* = 0.004) and ΔPMI/baseline (HR, 1.13; 95% CI, 1.09–1.17; *p* < 0.001) emerged as independent predictors of all‐cause mortality.

**TABLE 3:** Univariate and multivariate Cox proportional hazards regression analyses for all‐cause mortality and MACEs.

Variable	All‐cause mortality						MACEs					
	Univariate cox model			Multivariate cox model			Univariate cox model			Multivariate cox model		
	HR	95% CI	*p*	HR	95% CI	*p*	HR	95% CI	*p*	HR	95% CI	*p*
Demographics												
Age (per 1 year increment)	1.07	1.02–1.12	0.003	1.01	0.96–1.06	0.663	1.07	1.00–1.13	0.025	1.00	0.94–1.07	0.950
Male sex	2.19	0.87–5.50	0.096	1.31	0.42–4.09	0.643	0.70	0.32–1.56	0.387	0.45	0.18–1.12	0.086
Body mass index (per 1 kg/m^2^ increment)	0.89	0.82–0.97	0.009	1.12	0.94–1.33	0.198	0.94	0.85–1.04	0.247			
ASA score ≥ 3	1.21	0.88–4.02	0.210				1.11	0.85–3.02	0.240			
AAA diameter (per 1 mm increment)	1.04	1.02–1.06	< 0.001	1.02	0.99–1.05	0.308	1.02	0.99–1.05	0.154			
Comorbidities												
Hypertension	0.85	0.47–1.52	0.577				1.72	0.75–3.95	0.201			
Dyslipidemia	0.67	0.37–1.22	0.191				0.93	0.47–1.87	0.842			
Diabetes mellitus	1.19	0.61–2.31	0.613				2.72	1.35–5.50	0.005	2.54	1.21–5.36	0.014
Coronary artery disease	1.08	0.60–1.96	0.793				0.73	0.33–1.62	0.444			
Peripheral arterial disease	1.77	0.70–4.46	0.227				1.02	0.24–4.27	0.976			
Chronic obstructive pulmonary disease	1.34	0.67–2.66	0.411				1.44	0.62–3.30	0.394			
Stroke or transient ischemic attack	1.02	0.51–2.03	0.963				1.54	0.72–3.31	0.265			
Chronic kidney disease	1.58	0.88–2.85	0.127				3.16	1.60–6.23	< 0.001	2.44	1.12–5.33	0.025
Ever smoker	1.32	0.69–2.52	0.399				0.94	0.45–1.96	0.859			
GNRI	0.94	0.91–0.97	< 0.001	0.95	0.89–1.00	0.088	0.96	0.93–0.99	0.029	0.97	0.93–1.02	0.220
Perioperative parameters												
Operation time (per 1 min increment)	1.00	0.99–1.01	0.147				1.00	0.99–1.01	0.785			
Anesthesia time (per 1 min increment)	1.00	0.99–1.01	0.133				1.00	0.99–1.01	0.824			
Intraoperative blood loss (per 1 mL increment)	1.00	0.0.99–1.00	0.407				1.00	0.0.99–1.00	0.831			
Need for transfusion	1.50	0.36–6.19	0.575				2.27	0.54–9.53	0.263			
IIA embolization	0.85	0.47–1.53	0.584				0.80	0.38–1.68	0.558			
IMA embolization	1.30	0.61–2.76	0.495				0.95	0.34–2.71	0.929			
Post‐operative delirium	1.79	0.76–4.21	0.184				2.62	1.00–6.86	0.049	1.44	0.49–4.28	0.508
Time to independent ambulation (per 1 day increment)	1.38	1.21–1.56	< 0.001	1.25	1.07–1.46	0.007	1.32	1.14–1.54	< 0.001	1.19	1.00–1.42	0.048
Length of hospital stay (per 1 day increment)	1.04	1.01–1.07	0.009	1.03	0.95–1.12	0.490	0.99	0.91–1.07	0.761			
Pre‐operative sarcopenia	2.76	1.17–6.48	0.020	1.92	0.68–5.38	0.215	1.11	0.50–2.45	0.803			
ΔPMI/baseline (per 1% decrease)	1.13	1.10–1.16	< 0.001	1.13	1.09–1.17	< 0.001	1.09	1.05–1.13	< 0.001	1.10	1.06–1.15	< 0.001

Abbreviations: AAA, abdominal aortic aneurysms; ASA, american society of anesthesiologists; CI, confidence interval; GNRI, the geriatric nutritional risk index; HR, hazard ratio; IIA, internal iliac artery; IMA, inferior mesenteric artery; MACEs, major adverse cardiovascular events; PMI, psoas muscle index.

For MACEs, univariate analysis identified age, diabetes mellitus, chronic kidney disease, GNRI, post‐operative delirium, time to independent ambulation, and ΔPMI/baseline as significant factors. Multivariate analysis revealed that diabetes mellitus (HR, 2.54; 95% CI, 1.21–5.36; *p* = 0.014), chronic kidney disease (HR, 2.44; 95% CI, 1.12–5.33; *p* = 0.025), time to independent ambulation (HR, 1.19; 95% CI, 1.00–1.42; *p* = 0.048), and ΔPMI/baseline (HR, 1.10; 95% CI, 1.06–1.15; *p* < 0.001) were independent predictors of MACEs.

### 3.4 ROC Curve Analysis for All‐Cause Mortality

ROC curve analysis of time to independent ambulation and ΔPMI/baseline, both identified as significant predictors of all‐cause mortality in the multivariate Cox proportional hazards regression model, showed that the cut‐off value for time to independent ambulation was 2 days, with an area under the curve (AUC) of 0.58 (95% CI, 0.51–0.65), sensitivity of 0.31, and specificity of 0.83. The cut‐off value for ΔPMI/baseline was a decrease of 6.09%, with an AUC of 0.86 (95% CI, 0.80–0.93), sensitivity of 0.87, and specificity of 0.78.

Patients were classified into four groups based on the cut‐off values for time to independent ambulation (2 days) and ΔPMI/baseline (6.09% decrease). Group A, defined as time to independent ambulation < 2 days and ΔPMI/baseline with a decrease of < 6.09%, encompassed 115 patients. Group B, characterized by time to independent ambulation ≥ 2 days and ΔPMI/baseline with a decrease of < 6.09%, comprised 29 patients. Group C, defined by time to independent ambulation < 2 days and ΔPMI/baseline with a decrease of ≥ 6.09%, included 63 patients. Group D, with time to independent ambulation ≥ 2 days and ΔPMI/baseline with a decrease of ≥ 6.09%, comprised 21 patients. Comparisons of clinical background factors among the four groups revealed significant differences in age, sex, chronic kidney disease, GNRI, post‐operative delirium, and length of hospital stay (Table S1).

### 3.5 Kaplan–Meier Curves for All‐Cause Mortality and MACEs

**FIGURE 2:**
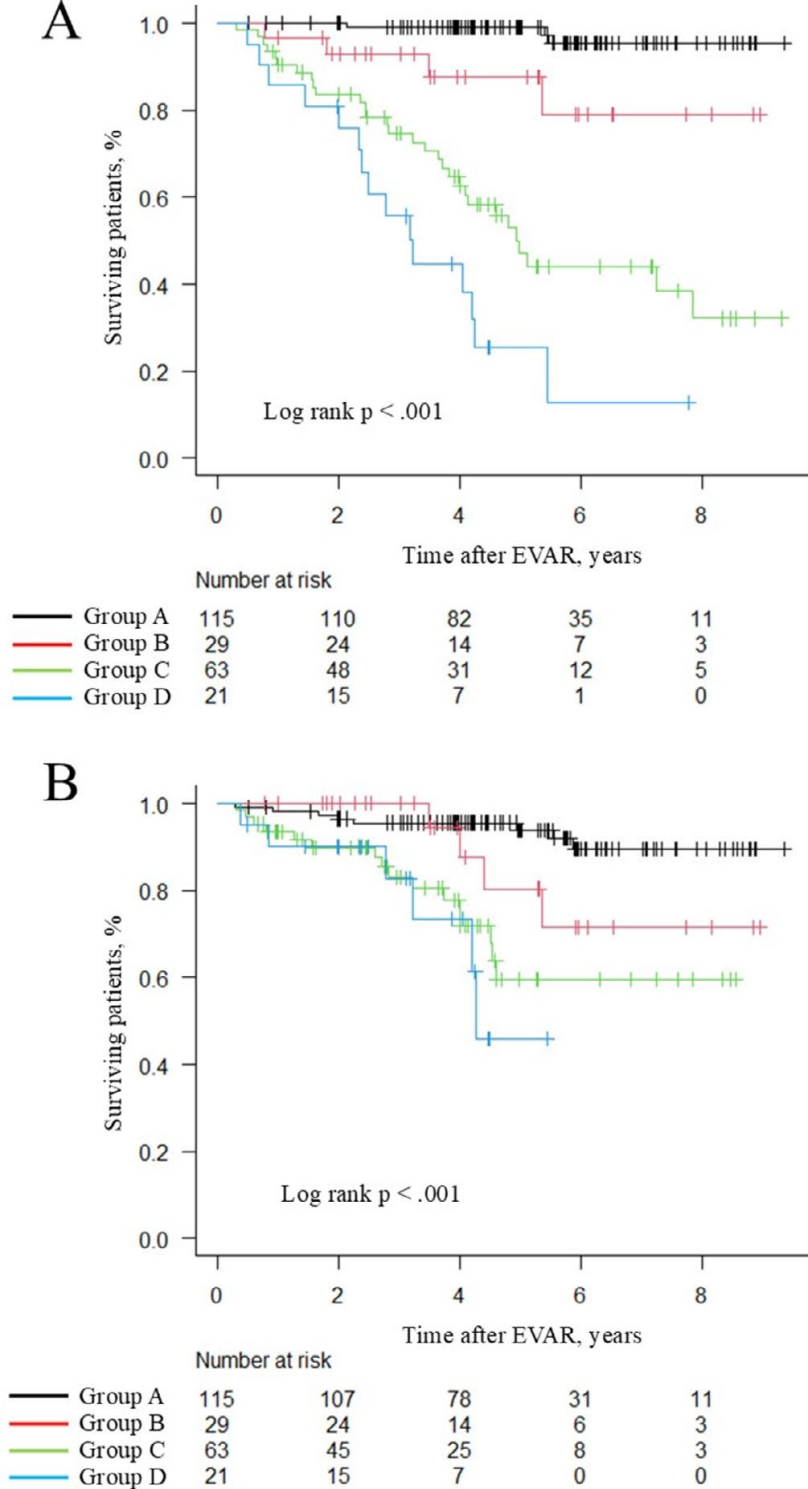
Kaplan–Meier curves for all‐cause mortality and MACEs grouped into four groups based on cut‐off values. (A) all‐cause mortality. (B) MACEs.

The Kaplan–Meier curves for all‐cause mortality and MACE stratified by these four groups are depicted in Figure 2. Regarding the primary endpoint of all‐cause mortality, Group D demonstrated the poorest survival. The 5‐year survival rates were 99.1% ± 1.6%, 87.7% ± 7.7%, 47.2% ± 7.3% and 25.5% ± 9.9% in Groups A, B, C, and D, respectively (log rank *p* < 0.001). Moreover, compared with the other groups, Group D exhibited the highest incidence of the secondary endpoint, MACEs (log rank *p* < 0.001).

### 3.6 Factors Associated With a ≥ 6.09% Decrease in ΔPMI/Baseline

Univariate and multivariate logistic regression analyses for factors associated with a ≥ 6.09% decrease in ΔPMI/baseline are presented in Table S2. In the multivariate analysis, chronic kidney disease was identified as an independent associated factor.

## 4 Discussion

To the best of our knowledge, this study is the first to evaluate the combined prognostic impact of delayed recovery of independent ambulation and sarcopenia progression in patients undergoing EVAR. Our findings demonstrate that both are independent predictors of all‐cause mortality and MACEs, and that their coexistence is associated with significantly worse outcomes. These results underscore the significance of perioperative physical function and muscle mass preservation to improve long‐term prognosis following EVAR.

Although no previous studies have specifically investigated gait acquisition in patients undergoing EVAR, 50 patients (21.9%) in the present cohort failed to achieve independent ambulation by post‐operative day 1, indicating delayed recovery. In contrast, previous studies on older adults undergoing cardiac surgery have reported higher rates of delayed ambulation, with 39.4%–47.5% of them unable to regain independent walking [19, 20]. Although definitions of independent ambulation differ, the lower incidence observed following EVAR suggests its less invasive nature than cardiac surgery. This study identified delayed recovery of ambulation as an independent predictor of all‐cause mortality and MACEs. Although the number of patients with delayed ambulation was relatively small, this finding suggests that it should be regarded as a clinically significant factor. Previous literature has demonstrated an association between reduced physical function due to delayed ambulation and worsening frailty scores, decreased frailty‐related instrumental activities of daily living and impaired mobility, persisting even after discharge [21]. Frailty progression is a strong prognostic factor in patients undergoing cardiovascular surgery [22, 23, 24]. Therefore, delayed ambulation contributing to frailty progression and subsequent adverse outcomes is plausible. The current results align with findings in patients undergoing cardiac surgery, wherein delayed ambulation has similarly been associated with poor prognosis [6]. However, it should be noted that the ROC analysis for time to independent ambulation yielded a relatively low AUC (0.56), indicating limited discriminative power for predicting outcomes. This suggests that assessment of delayed ambulation alone may be insufficient and combining it with other indicators.

In this study, the PMI significantly decreased from the baseline to 6 months post‐operatively, indicating a relatively short period of decline. Moreover, chronic kidney disease was identified as an independent risk factor for a greater decrease in PMI. Chronic kidney disease has been reported to promote the progression of sarcopenia through metabolic and hormonal dysregulation, including uraemia and hyperparathyroidism, as well as low‐grade systemic inflammation. These mechanisms may support the findings of the present study [25]. Among patients undergoing EVAR, previous studies reported that PMA significantly declined over an average follow‐up of 207 days, consistent with our findings [4]. The age‐dependent loss of skeletal muscle mass in healthy Asian adults is approximately 0.5%–1% per year [26], whereas the mean observed 6‐month ΔPMI/baseline in our cohort was 4.5%, indicating a markedly accelerated decline after EVAR. The underlying mechanisms for this accelerated decline cannot be determined from the present study. However, previous studies have suggested that impaired lower‐limb perfusion after EVAR [4] and the high prevalence of pre‐existing sarcopenia in this population may predispose patients to more pronounced skeletal muscle loss. The observed decrease in ΔPMI/baseline, indicating sarcopenia progression, may in turn contribute to a negative cycle, known as the frailty cycle, characterized by reduced physical activity and malnutrition [27]. Overall, these findings suggest that decreased ΔPMI/baseline can be linked to poor prognosis.

Previous studies have indicated that pre‐operatively measured PMA is associated with late survival in patients undergoing EVAR or open repair for AAA [9]. However, they have not concentrated on post‐operative changes in muscle size. In our analysis, the baseline PMI was not significantly associated with mortality when the dynamic changes in the PMI were considered. To our knowledge, Lindström et al. [28] are the only researchers who have investigated the association between dynamic changes in skeletal muscle mass following EVAR and patient prognosis. In our cohort, over 75% of patients had pre‐operative sarcopenia, which may have limited the variability of baseline PMI and contributed to its lack of significance as an independent predictor. Nevertheless, consistent with Lindström et al., baseline skeletal muscle mass was not associated with prognosis, whereas dynamic changes were significantly associated with outcomes, in line with their findings. However, in their study, 31.1% of baseline CT images were obtained within 1 month post‐operatively, and the average interval to follow‐up CT was 1.9 years, which is relatively long. In contrast, in our study, baseline CT images were obtained within a short period before surgery in all patients (mean, 1.1 ± 0.9 months pre‐operatively), facilitating more sensitive detection of skeletal muscle changes over the relatively short 6‐month post‐operative period. Considering that skeletal muscle mass changes can develop as early as 6 months following EVAR, the findings of this study may provide clinical advantages by enabling preventive measures to be performed at an earlier stage. Furthermore, chronic kidney disease was identified as an independent risk factor for accelerated sarcopenic progression. This finding may be clinically useful for preoperative risk stratification, enabling earlier identification of patients who are at high risk of postoperative muscle loss and may benefit from targeted interventions.

Considering the substantial number of reinterventions frequently required following EVAR [29], careful evaluation of long‐term survival is warranted. In this context, delayed recovery of ambulation and dynamic changes in relative PMI should be recognized as significant prognostic indicators. Their potential value as novel markers of poor survival warrants further investigation, particularly when considered alongside other major determinants of perioperative and postoperative outcomes.

The role of rehabilitation following EVAR should not be underestimated considering the association between delayed ambulation, post‐operative muscle loss and adverse outcomes. Early mobilization has been shown to prevent post‐operative complications, enhance functional capacity, and reduce the length of hospital stay among older patients undergoing cardiac surgery [30]. Furthermore, multimodal interventions, including resistance training and nutritional support, have proven effective in preventing or reversing sarcopenia across various clinical settings [31, 32]. Tailored rehabilitation protocols for patients undergoing EVAR, particularly those at risk of delayed recovery, may contribute to better functional recovery and enhanced long‐term prognosis.

This study had several limitations. First, as this was a retrospective observational study, whether accelerating post‐operative gait recovery or preventing sarcopenia progression would lead to improved prognosis could not be determined. Therefore, prospective interventional studies are warranted. Second, we were unable to monitor patients' exercise habits and nutritional status following hospital discharge. These factors may also impact sarcopenia progression and overall mortality. Third, assessments of sarcopenia and frailty were limited. Sarcopenia was evaluated solely based on the PMI, which may not fully capture its complex nature. Moreover, postoperative reduction in psoas muscle volume may partly reflect the effects of surgery itself rather than true sarcopenic progression, and thus may not accurately represent overall skeletal muscle mass. In addition, standardized frailty indices were not incorporated. Although time to independent ambulation and sarcopenia progression can reflect certain aspects of frailty, the inclusion of validated frailty scores and functional assessments, such as muscle strength and physical performance, would provide a more comprehensive evaluation of patients' vulnerability. Finally, as in most studies on AAA, females were underrepresented owing to the epidemiology of the condition, which may limit the generalizability of the findings to female patients.

## 5 Conclusions

In conclusion, delayed recovery of independent ambulation and post‐operative sarcopenia progression emerged as independent predictors of all‐cause mortality and MACEs following EVAR. Notably, pre‐operative sarcopenia was not a significant prognostic factor, suggesting that muscle loss progression following surgery can have a greater impact on long‐term outcomes than pre‐existing muscle depletion. Furthermore, patients who experienced delayed ambulation recovery (≥ 2 days) and a decline in PMI/BL of > 6.09% were identified as being at particularly high risk. These results propose a novel prognostic framework for patients undergoing EVAR, highlighting the potential role of early rehabilitation strategies for improving outcomes.

## Author Contributions

**Hirokazu Sugiura:** conception and design, data collection, analysis and interpretation of data, drafting of the manuscript, approval of the final version, and agreement to be accountable for all aspects of the work. **Tsuyoshi Shibata:** conception and design, analysis and interpretation of data, drafting of the manuscript, critical revision, approval of the final version, and agreement to be accountable for all aspects of the work. **Yutaka Iba:** conception and design, analysis and interpretation of data, critical revision, approval of the final version, and agreement to be accountable for all aspects of the work. **Shingo Tsushima**, **Kenta Yoshikawa**, and **Shun Hayasaka:** conception and design, data collection, analysis and interpretation of data, critical revision. **Tomohiro Nakajima**, **Junji Nakazawa**, **Ayaka Arihara**, **Kenichi Kato**, **Shigeki Komatsu**, **Masato Yonemori**, **Hajime Maeda**, **Masanori Nakamura**, **Yuki Sugawara**, and **Nobuyoshi Kawaharada:** conception and design, and critical revision.

## Funding

The authors have nothing to report.

## Ethics Statement

This study was conducted in accordance with the ethical principles of the Declaration of Helsinki and was approved by the Human Investigation Committee of Sapporo City General Hospital (approval number: R06–064–1141) and Sapporo Medical University (approval number: 162–140). Informed consent was obtained using an opt‐out approach via the hospital's official website.

## Consent

The requirement for individual informed consent was waived due to the retrospective design of the study, which was approved by the institutional review board.

## Conflicts of Interest

The authors declare no conflicts of interest.

## Supporting information


**Figure S1:** Cross‐sectional area of the psoas muscle at the third lumbar vertebral level.

**Figure S2:** Histogram of the time to independent ambulation.

**Figure S3:** (A) Histogram of PMI at baseline. (B) Histogram of ΔPMI/baseline.

**Table S1:** Comparisons of clinical background characteristics among the four patient groups.
**Table S2:** Univariate and multivariate logistic regression analyses of factors associated with a ≥ 6.09% decrease in ΔPMI/baseline.

## Data Availability

The data that support the findings of this study are available from the corresponding author upon reasonable request.
