# Supplementary figures and images for: Impact of Delayed Recovery of Independent Ambulation and Sarcopenia Progression on Long‐Term Outcomes Following Endovascular Aortic Aneurysm Repair
Source: Geriatr Gerontol Int. 2026 Jan 21;26(1):e70355. doi: 10.1111/ggi.70355 (PMC12824468; doi:10.1111/ggi.70355)

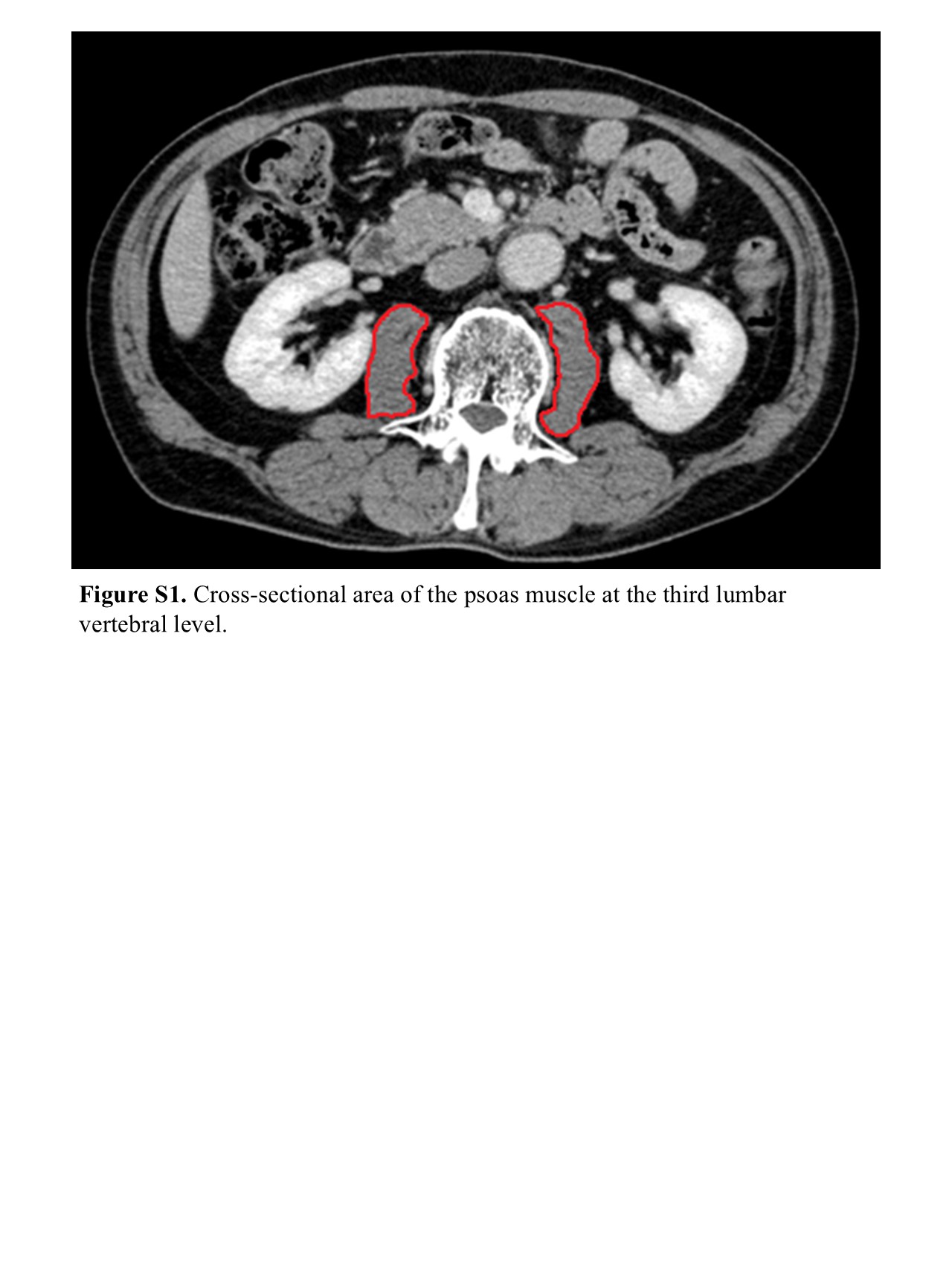

Supplement: Supplementary file 1 — Figure S1: Cross‐sectional area of the psoas muscle at the third lumbar vertebral level. [file GGI-26-0-s003.jpg]

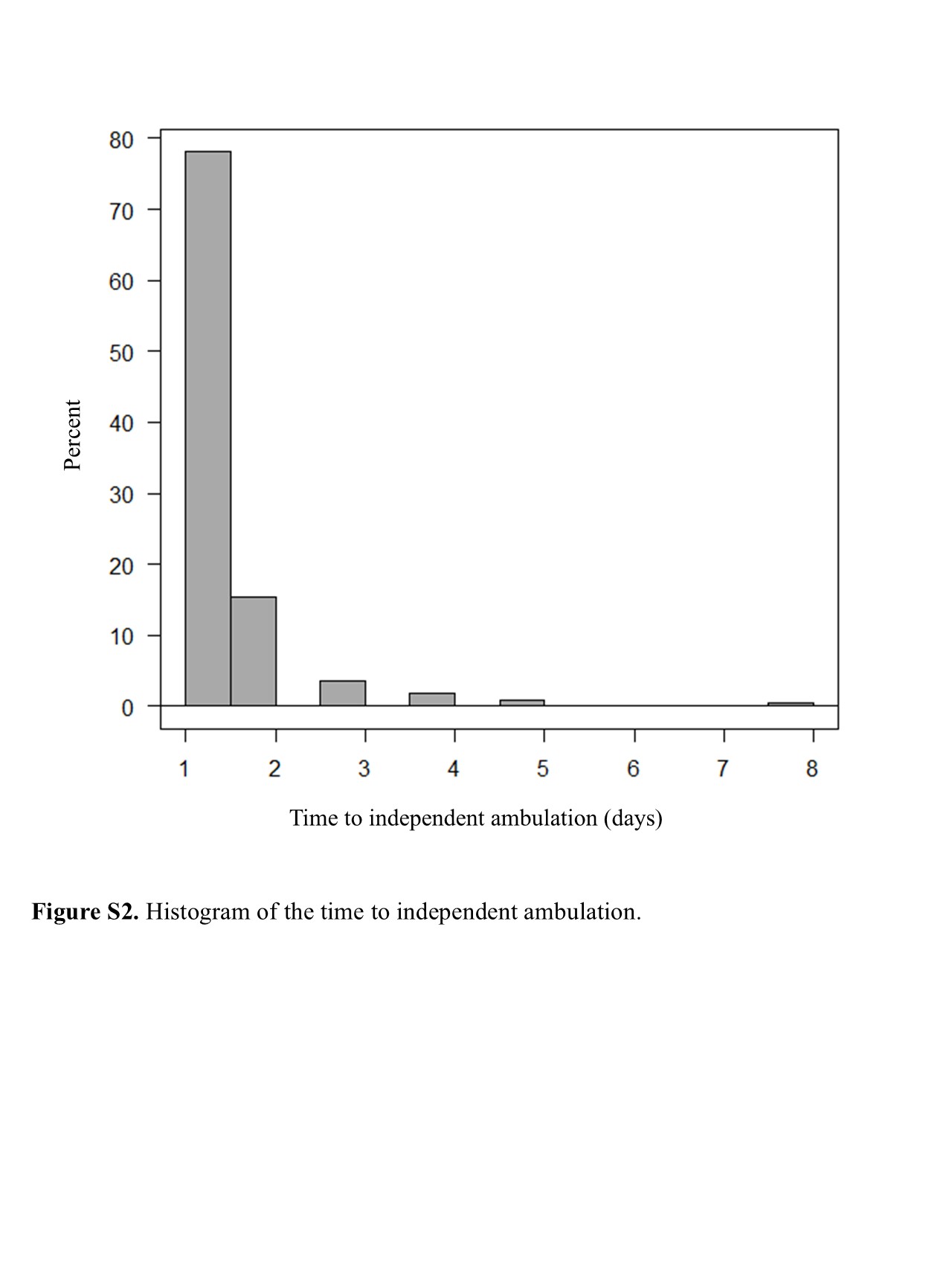

Supplement: Supplementary file 2 — Figure S2: Histogram of the time to independent ambulation. [file GGI-26-0-s001.jpg]

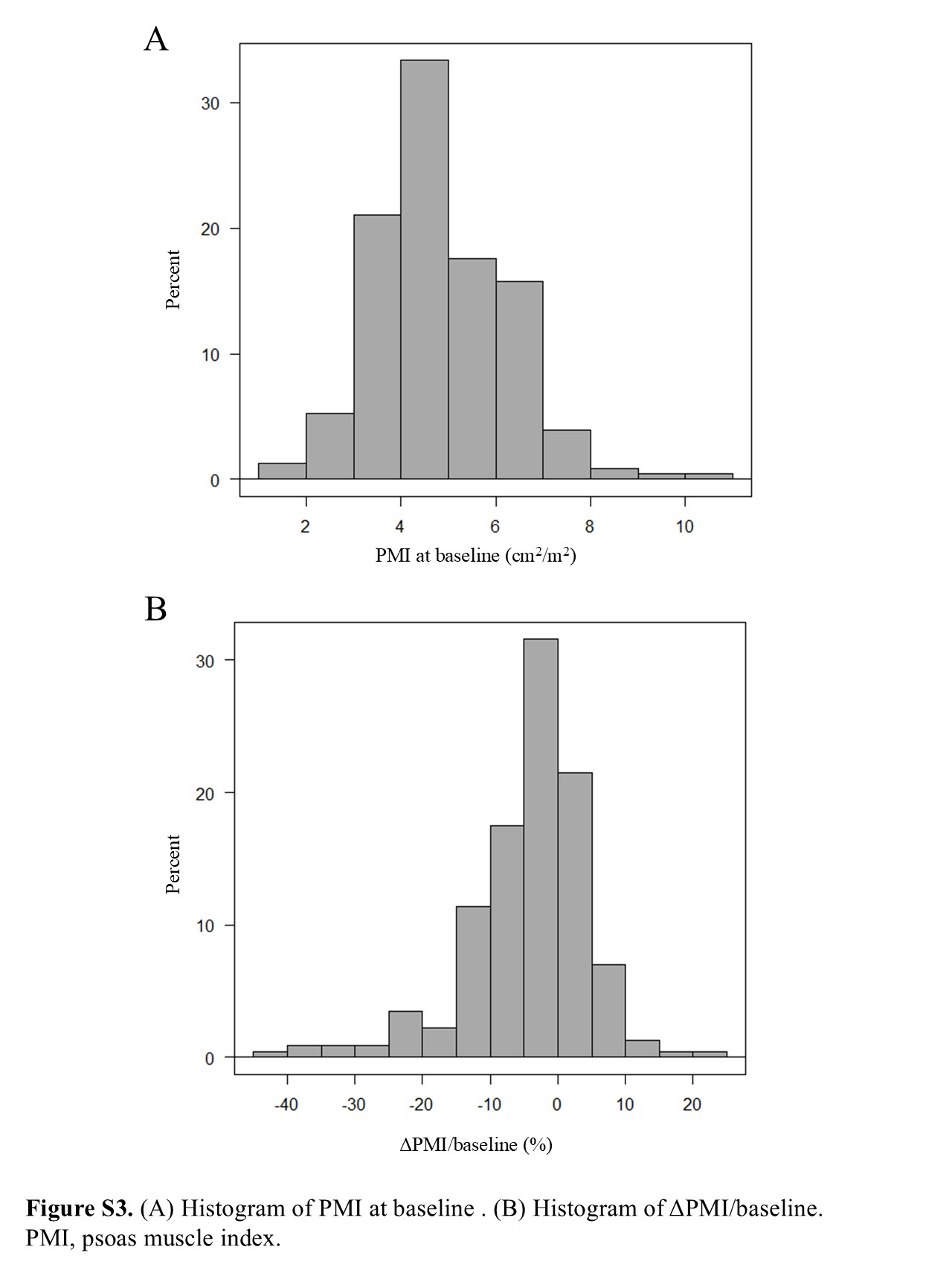

Supplement: Supplementary file 3 — Figure S3: (A) Histogram of PMI at baseline. (B) Histogram of ΔPMI/baseline. [file GGI-26-0-s004.jpg]
