# Supplementary material for: Impact of Delayed Recovery of Independent Ambulation and Sarcopenia Progression on Long‐Term Outcomes Following Endovascular Aortic Aneurysm Repair
Source: Geriatr Gerontol Int. 2026 Jan 21;26(1):e70355. doi: 10.1111/ggi.70355 (PMC12824468; doi:10.1111/ggi.70355)
Supplement: Supplementary file 4 — Table S1: Comparisons of clinical background characteristics among the four patient groups. Table S2: Univariate and multivariate logistic regression analyses of factors associated with a ≥ 6.09% decrease in ΔPMI/baseline. [file GGI-26-0-s002.docx]

**Table S1** Comparisons of clinical background characteristics among the four patient groups

| Variable | Group A (n = 115) | Group B (n = 29) | Group C (n = 63) | Group D (n = 21) | P-value |  |  |
| --- | --- | --- | --- | --- | --- | --- | --- |
| *Demographics* |  |  |  |  |  |  |  |
| Age, years | 76. 9 ± 6.2 | 80.8 ± 6.2 | 78.0 ± 6.6 | 81.5 ± 6.7 | .002 |  |  |
| Male | 93 (80.9) | 18 (62.1) | 58 (92.1) | 19 (90.5) | .004 |  |  |
| Body mass index, kg/m2 | 24.1 ± 3.6 | 23.7 ± 3.9 | 22.7 ± 2.5 | 23.7 ± 4.2 | .106 |  |  |
| ASA score ≥ 3 | 61 (53.0) | 15 (51.7) | 32 (50.8) | 12 (57.1) | .802 |  |  |
| AAA diameter, mm | 48.4 ± 11.5 | 48.9 ± 9.0 | 51.2 ± 10.9 | 54.0 ± 10.5 | .122 |  |  |
| *Comorbidities* |  |  |  |  |  |  |  |
| Hypertension | 79 (68.7) | 19 (65.5) | 44 (69.8) | 13 (61.9) | .905 |  |  |
| Dyslipidemia | 37 (32.2) | 17 (58.6) | 22 (34.9) | 7 (33.3) | .065 |  |  |
| Diabetes mellitus | 13 (11.3) | 6 (20.7) | 18 (28.6) | 2 (9.5) | .059 |  |  |
| Coronary artery disease | 36 (31.3) | 8 (27.6) | 22 (34.9) | 6 (28.6) | .889 |  |  |
| Peripheral arterial disease | 6 (5.2) | 2 (6.9) | 7 (11.1) | 0 (0.0) | .268 |  |  |
| Chronic obstructive pulmonary disease | 16 (13.9) | 6 (20.7) | 10 (15.9) | 2 (9.5) | .710 |  |  |
| Stroke or transient ischemic attack | 19 (16.5) | 8 (27.6) | 12 (19.0) | 5 (23.8) | .547 |  |  |
| Chronic kidney disease | 14 (12.2) | 11 (37.9) | 17 (27.0) | 8 (38.1) | .002 |  |  |
| Ever smoker | 87 (75.7) | 16 (55.2) | 47 (74.6) | 12 (57.1) | .069 |  |  |
| GNRI | 104.5 ± 9.8 | 102.0 ± 9.7 | 100.5 ± 8.5 | 101.5 ± 9.0 | .043 |  |  |
| *Perioperative parameters* |  |  |  |  |  |  |  |
| Operation time, min | 132.6 ± 59.3 | 145.1 ± 49.9 | 139.1 ± 47.1 | 165.0 ± 48.8 | .082 |  |  |
| Anesthesia time, min | 196.9 ± 63.0 | 211.6 ± 55.2 | 207.9 ± 50.5 | 229.5 ± 56.1 | .094 |  |  |
| Intraoperative blood loss, mL | 69.1 ± 134.0 | 52.1 ± 88.1 | 63.8 ± 106.0 | 102.9 ± 115.3 | .502 |  |  |
| Need for transfusion | 5 (4.3) | 0 (0.0) | 1 (1.6) | 1 (4.8) | .529 |  |  |
| IIA embolization | 41 (35.7) | 12 (41.4) | 21 (33.3) | 4 (19.0) | .402 |  |  |
| IMA embolization | 17 (14.8) | 2 (6.9) | 3 (4.8) | 4 (19.0) | .119 |  |  |
| Post-operative delirium | 5 (4.3) | 8 (27.6) | 6 (9.5) | 2 (9.5) | .002 |  |  |
| Length of hospital stay, days | 8.7 ± 2.9 | 11.2 ± 9.1 | 8.5 ± 1.8 | 14.1 ± 11.1 | < .001 |  |  |
| Pre-operative sarcopenia | 89 (77.4) | 19 (65.5) | 51 (81.0) | 17 (81.0) | .404 |  |  |

Data are presented as n (%), mean ± standard deviation. ASA, american society of anesthesiologists; AAA, abdominal aortic aneurysms; GNRI, the geriatric nutritional risk index; IIA, internal iliac artery; IMA, inferior mesenteric artery

**Table S2** Univariate and multivariate logistic regression analyses of factors associated with a ≥6.09% decrease in ΔPMI/baseline

| Variable | Univariate model | | | Multivariate model | | |
| --- | --- | --- | --- | --- | --- | --- |
|  | OR | 95% CI | P-value | OR | 95% CI | P-value |
| *Demographics* |  |  |  |  |  |  |
| Age (per 1 year increment) | 1.03 | 0.99 –1.07 | .188 |  |  |  |
| Male sex | 1.27 | 0.87 – 2.50 | .096 |  |  |  |
| Body mass index (per 1 kg/m^2^ increment) | 0.91 | 0.84 – 0.99 | .034 | 0.96 | 0.84 – 1.11 | .588 |
| ASA score ≥ 3 | 1.21 | 0.88 – 3.02 | .202 |  |  |  |
| AAA diameter (per 1 mm increment) | 1.03 | 1.01 – 1.05 | .052 |  |  |  |
| *Comorbidities* |  |  |  |  |  |  |
| Hypertension | 0.99 | 0.56 – 1.76 | .975 |  |  |  |
| Dyslipidemia | 0.88 | 0.50 – 1.54 | .652 |  |  |  |
| Diabetes mellitus | 2.06 | 1.02 – 4.13 | .043 | 1.95 | 0.95 – 4.00 | .067 |
| Coronary artery disease | 1.14 | 0.64 – 2.02 | .663 |  |  |  |
| Peripheral arterial disease | 1.55 | 0.54 – 4.43 | .417 |  |  |  |
| Chronic obstructive pulmonary disease | 0.92 | 0.43 – 1.98 | .839 |  |  |  |
| Stroke or transient ischemic attack | 1.10 | 0.56 – 2.16 | .784 |  |  |  |
| Chronic kidney disease | 2.02 | 1.07 – 3.81 | .031 | 2.04 | 1.05 – 3.96 | .035 |
| Ever smoker | 0.94 | 0.52 – 1.70 | .836 |  |  |  |
| GNRI | 0.96 | 0.94 – 0.99 | .013 | 0.97 | 0.93 – 1.02 | .260 |
| *Perioperative parameters* |  |  |  |  |  |  |
| Operation time (per 1 mm increment) | 1.00 | 0.99 – 1.01 | .167 |  |  |  |
| Anesthesia time (per 1 mm increment) | 1.00 | 0.99 – 1.01 | .098 |  |  |  |
| Intraoperative blood loss (per 1 mL increment) | 1.00 | .0.99 – 1.00 | .632 |  |  |  |
| Need for transfusion | 0.68 | 0.13 – 3.57 | .647 |  |  |  |
| IIA embolization | 0.73 | 0.41 – 1.30 | .280 |  |  |  |
| IMA embolization | 0.60 | 0.24 – 1.49 | .269 |  |  |  |
| Post-operative delirium | 1.06 | 0.42 – 2.67 | .901 |  |  |  |
| Time to independent ambulation (per 1 day increment) | 1.29 | 0.95 – 1.76 | .101 |  |  |  |
| Length of hospital stay (per 1 day increment) | 1.02 | 0.97 – 1.08 | .354 | 1.03 | 0.95 – 1.12 | .490 |
| Pre-operative sarcopenia | 1.42 | 0.73 – 2.75 | .303 | 1.92 | 0.68 – 5.38 | .215 |

PMI, psoas muscle index; OR, odds ratio; CI, confidence interval; ASA, american society of anesthesiologists; AAA, abdominal aortic aneurysms; GNRI, the geriatric nutritional risk index; IIA, internal iliac artery; IMA, inferior mesenteric artery.
